# Multi-Objective Approach for Energy-Aware Workflow Scheduling in Cloud Computing Environments

**DOI:** 10.1155/2013/350934

**Published:** 2013-11-04

**Authors:** Sonia Yassa, Rachid Chelouah, Hubert Kadima, Bertrand Granado

**Affiliations:** ^1^L@RIS Laboratory, EISTI, Avenue du Parc, 95011 Cergy-Pontoise, France; ^2^ETIS Laboratory, CNRS UMR8051, University of Cergy-Pontoise, ENSEA, 6 Avenue du Ponceau, 95014 Cergy-Pontoise, France

## Abstract

We address the problem of scheduling workflow applications on heterogeneous computing systems like cloud computing infrastructures. In general, the cloud workflow scheduling is a complex optimization problem which requires considering different criteria so as to meet a large number of QoS (Quality of Service) requirements. Traditional research in workflow scheduling mainly focuses on the optimization constrained by time or cost without paying attention to energy consumption. The main contribution of this study is to propose a new approach for multi-objective workflow scheduling in clouds, and present the hybrid PSO algorithm to optimize the scheduling performance. Our method is based on the Dynamic Voltage and Frequency Scaling (DVFS) technique to minimize energy consumption. This technique allows processors to operate in different voltage supply levels by sacrificing clock frequencies. This multiple voltage involves a compromise between the quality of schedules and energy. Simulation results on synthetic and real-world scientific applications highlight the robust performance of the proposed approach.

## 1. Introduction

Cloud computing presents an interesting technology that facilitates the execution of scientific and commercial applications. It provides, on demand, flexible and scalable services to customers through a pay per use basis. It can usually provide three kinds of services: IaaS (Infrastructure as a Service), PaaS (Platform as a Service), and SaaS (Software as a Service). These services are offered with different service levels so as to meet the needs of various customer groups. Although many cloud services have a similar functionality (e.g., computing services, storage services, network services, etc.), they differ from each other by non-functional qualities termed QoS (Quality of Service) parameters, such as service time, service cost, service availability, service energy consumption, service utilization, and so forth. 

These QoS parameters may be defined and proposed by different SLAs (Service Level Agreements). An SLA specifies the QoS requirements of negotiated resources, the minimum expectations and limits that exist between consumers and providers. Applying such an SLA represents a binding contract. Lack of such agreements can lead applications to move away from the cloud and will compromise the future growth of cloud computing.

Several scientific applications such as those of bioinformatics, chemistry and astronomy contain a great number of tasks that have precedence constraints. They can be defined by DAGs (Directed Acyclic Graph). These scientific workflows typically involve complex data of different sizes and long term computer simulations. They need high computation power and the availability of large infrastructures that grid and more recently cloud computing environments provide with different QoS levels.

Due to the importance of workflow applications, several research projects have been conducted to develop workflow management systems with scheduling algorithms. The projects: Condor Dagman [1], Gridbus toolkit [2], Iceni [3], Pegasus [4], and so forth, are designed for grids, whereas cloudbus toolkit [5], SwinDeW-C [6], VGrADS [7], and so forth, are developed for clouds. These systems can be viewed as a type of platform service facilitating the automation of scientific and commercial applications on the grid and cloud by masking their orchestrations and executions. 

In order to effectively schedule the tasks and data applications on these cloud environments, workflow management systems require more elaborated scheduling strategies to meet QoS constraints and the precedence relationships between workflow tasks. The study of workflow scheduling is becoming an important challenge in the area of cloud computing.

The workflow scheduling in the cloud is a difficult problem. This problem is even more difficult when there are several factors to be considered namely, (1) the various QoS requirements of customers like service response time, service cost, and so forth; (2) the heterogeneity, dynamicity and elasticity of cloud services; (3) the various ways of combining these services to execute workflow tasks; (4) the transfer of large volumes of data, and so forth. However, the workflow scheduling problem is seen as a combinatorial problem, where it is impossible to find the globally optimal solution by using simple algorithms or rules. It is well known as an NP-complete problem [8] and depends on the problem size.

The workflow scheduling problem has been widely studied in many previous works [9–12]. Most of these works have concentrated only on two QoS parameters namely, the deadline and budget. In this paper, we extend these works to handle multiple QoS requirements. We address the QoS-based workflow scheduling which aims to minimize the cost and total time execution of user applications as specified in the SLA. Furthermore, the scheduler must also be able to schedule workflow tasks so as to maximize the provider profits by minimizing energy consumption while preserving the users QoS preferences. We achieve this by using an iterative method called Multi-objective Discrete Particle Swarm Optimization (MODPSO) combined with the Dynamic Voltage and Frequency Scaling (DVFS) technique. This last one allows a compromise between system performance and energy consumption. 

The proposed approach is assessed by simulation runs on a set of synthetic and real-world scientific applications. Simulation results showed that this new multi-objective algorithm significantly improves the performance of related approaches.

The remainder of this paper is organized as follows.  Section 2 reviews several related works.  Section 3 presents the problem modeling of the QoS based workflow scheduling.  Section 4 describes in detail our scheduling heuristic called DVFS-MODPSO.  Section 5 shows an experimental evaluation of our heuristic.  Section 6 concludes the paper and discusses some future works.

## 2. Related Work

The workflow scheduling problem in heterogeneous computing systems is an NP-hard optimization problem [8], meaning that the amount of computation needed to find optimum solutions increases exponentially with the problem size. Previous works have proposed many heuristic, and meta-heuristic based approaches [13–16] to solve this problem. One of the most widely used heuristics for scheduling workflow application is the Heterogeneous Earliest Finish Time (HEFT) algorithm developed by Topcuoglu et al. [17]. HEFT is a static scheduling algorithm that attempts to minimize execution time (makespan). It preserves the workflow precedence constraints and produces a good schedule length.

Most of these previous works have focused on minimizing the workflow execution time without considering the users' budget constraint. However, with the market-oriented business model in cloud computing environments, where users are billed for their consumption of resources, several works that consider users' budget and deadline have been proposed [18–21]. In [22], a study indicating how to schedule scientific workflow applications with budget and deadline constraints onto computational grids using genetic algorithms is presented. Authors in [6] proposed an improved cost-based scheduling algorithm for making efficient scheduling of tasks to available resources in cloud. In [9], a particle swarm optimization (PSO) based heuristic is used to minimize the execution cost of scheduling workflow applications to cloud resources. 

Besides makespan and cost, energy consumption is becoming more and more important in the cloud computing environments. However, cloud providers must adopt measures not only to meet the user' QoS requirements, but also to ensure that their profit margin is not dramatically reduced due to high energy consumptions. The energy efficiency can conflict with the other QoS requirements (makespan, cost). Incorporating the energy consumption into the workflow scheduling adds another layer of complexity. Therefore, recent works have concentrated on developing energy-aware scheduling algorithms. They have examined various techniques such as dynamic power management, Dynamic Voltage and Frequency Scaling (DVFS) or resource hibernation [23–26]. Authors in [27] presented an online dynamic power management strategy with many power-saving states. They proposed a min-min based energy-aware scheduling algorithm to minimize energy consumption in heterogeneous computing systems. In [26], a dynamic slack allocation technique which tries to use idle (slack) time slots of processors to lower supply voltage (frequency/speed) is presented. These slack time slots occur, due to earlier completion and/or dependencies of tasks. Several DVS-based approaches for slack allocation have been proposed for both independent [28–35], and precedence-constrained [36–42] tasks. In [43], an energy-aware scheduling algorithm and detailed discussion of slack time computation are presented. This scheduling algorithm reduces voltages during the communication phases between parallel tasks on homogeneous processors. In [44], an Energy Conscious Scheduling heuristic (ECS) is proposed. The heuristic is devised with relative superiority as a novel objective function, which takes into account energy and makespan. ECS is used to improve the bi-objective genetic algorithm proposed in [45]. This latter has been extended in [46] to a parallel model of their approach. 

All of these presented works have focused on optimizing either a single or two objectives but none of them consider the relationships between several objectives, namely, the relationship between energy, makespan and cost. They do not take into account how each one of these criteria can affect others. To deal with these misses, we propose a Multi-Objective Discrete Particle Swarm Optimization algorithm combined with DVFS technique (DVFS-MODPSO) to optimize all three objectives at the same time. Our new approach provides a set of solutions named Pareto solutions (i.e. non-dominated solutions) enabling the user to select the desired tradeoff.

To the best of our knowledge, none of the previous scheduling approaches deal with the three-dimensional makespan/cost/energy optimization, when tackling the problem of scheduling workflow applications on heterogeneous computing environments such as the cloud computing ones, which constitute our key novelties.

## 3. Problem Modeling

In this section, we describe our system model in a formal way. Our ultimate goal is to distribute workflow tasks among cloud services so as to optimize both the users' QoS criteria and cloud providers' profits by saving energy consumption of their services. Therefore, we first present the cloud computing model. Then, we describe our workflow model and the QoS parameters we deal. We conclude this section by describing the scheduling model formalized as a multi-objective optimization problem we solve.

### 3.1. Cloud Computing Model

The cloud computing system used in this work is a set of resources offered by a cloud provider to run client applications. Our cloud model is inspired by the model described in [45]. We assume that the cloud is hosted in data centers composed of heterogeneous machines. These data centers deliver a variety of services hosted on thousands of IT servers, which are made available as subscription-based services in a pay-as-you-go model. In our model, the cloud computing system consists of a set of *P* = {*p*_1_, *p*_2_,…, *p*_*m*_} heterogeneous processors which are fully interconnected. The processors have varied processing capability delivered at different processing prices (see ec of Table 1). Each processor *p*_*j*_ ∈ *P* is DVFS-enabled; that is, it can operate with different VSLs (Voltage Scaling Level, i.e., different clock frequencies). For each processor *p*_*j*_ ∈ *P*, a set *V*_*j*_ of *v* VSLs is randomly and uniformly distributed among three different sets of VSLs (Table 1). We consider that processors consume energy during periods of inactivity; that is, when a processor is idling, it is assumed that the lowest voltage is supplied [44]. Because clock frequency transition overheads usually take a negligible amount of time, these overheads are not considered in this paper and the inclusion of such an overhead will have no bearing on the overall model of the proposed study.

Additionally, each processor *p*_*j*_ ∈ *P* has a set of links *Lp*_*j*_ = {*l*_*j*_, *p*_1_, *l*_*j*_, *p*_2_,…, *l*_*j*_, *p*_*k*_}, 1 ≤ *k* ≤ *m*; where *l*_*j*,*i*_ ∈ *R* ^+^ is the available bandwidth—measured in Mega bits per second (Mbps)—in the link between processors *p*_*j*_ and *p*_*i*_, with *l*_*j*,*j*_ = 1. We assume that a message can be transmitted from one processor to another while a task is executed on the recipient processor. Finally, communication between tasks executed on the same processor is neglected. Table 1 shows DVFS levels, Relative speeds (R.Speed) and execution costs (ec.) for three processor classes (TURION MT-34, OMAP, PENTIUM M).

### 3.2. Workflow Application Model

We model a cloud workflow application as a Directed Acyclic Graph (DAG), denoted as *G*(*V*, *E*). The set of nodes *V* = {*T*_1_,…, *T*_*n*_} represents the tasks in the workflow application, the set of arcs denotes precedence constraints and the control/data dependencies between tasks. An arc is in the form of *d*_*ij*_ = (*T*_*i*_, *T*_*j*_) ∈ *E*, where *T*_*i*_ is called the parent task of *T*_*j*_, *T*_*j*_ is the child task of *T*_*i*_, *d*_*ij*_ is the data produced by *T*_*i*_ and consumed by *T*_*j*_. We assume that a child task cannot be executed until all of its parent tasks have been completed. In a given task graph, a task with no parent is referred as an *entry task*, and one without any child is called an *exit task*. Since our algorithm involves only one entry and one exit tasks, we add two dummy tasks *T*_entry_ and *T*_exit_ which have zero execution time to the beginning and the end of the workflow, respectively. These dummy tasks are connected with zero-weight arcs to the actual entry and exit tasks, respectively.

We assume that each task *T*_*i*_ ∈ *V* has an associated basic execution time which is an independent value for each machine. We denote *w*_*ij*_, the basic computation time of a task *T*_*i*_ on a compute resource *p*_*j*_ at maximum speed and voltage (i.e., it corresponds to Level 1 in Table 1). The average execution time of the task *T*_*i*_ is defined as:

(1) $$\begin{array}{c} {\bar{w}}_{i}=\sum_{j=1}^{m}\;\frac{w_{ij}}{m}. \end{array}$$

Real computation time *wr*_*ijk*_ of the task *T*_*i*_ on machine *p*_*j*_ using relative execution speed *s*_*kj*_ is defined as:

(2) $$\begin{array}{c} {wr}_{ijk}=\frac{w_{ij}}{s_{kj}}. \end{array}$$

We also assume that every edge (*T*_*i*_, *T*_*j*_) ∈ *E*, is associated with value tr⁡_*ij*_, representing the time needed to transfer data from *T*_*i*_ to *T*_*j*_. The transfer time can be calculated according to the bandwidth *l*_*x*,*y*_ between the resources executing these tasks (*p*_*x*_ and *p*_*y*_ resp.) as follows:

(3) $$\begin{array}{c} {\mathrm{tr}}_{ij}=\frac{d_{ij}}{l_{x,y}}. \end{array}$$

However, a communication time is only required when two tasks are assigned to different processors. That is, the communication time when tasks are assigned to the same processor can be neglected, that is, 0. 

In general the execution costs (ec) and transmission costs (trc) are inversely proportional to the execution times and transmission times respectively.

We define *pred *(*T*_*i*_) as the set of all predecessors of *T*_*i*_ and *succ *(*T*_*i*_) as the set of all successors of *T*_*i*_. An ancestor of node *T*_*i*_ is any node *T*_*k*_ that is contained in *pred *(*T*_*i*_), or any node *T*_*j*_ that is also an ancestor of any node *T*_*k*_ contained in *pred *(*T*_*k*_). 

The Earliest Start Time and the Earliest Finish Time of a task *T*_*i*_ on a processor *p*_*j*_ are represented as *EST *(*T*_*i*_, *p*_*j*_) and *EFT *(*T*_*i*_, *p*_*j*_), respectively. *EST *(*T*_*i*_) and *EFT *(*T*_*i*_) represent the earliest start and finish times on any processor respectively. 

*P*_*av*(*T*_*i*_,*p*_*j*_)_ is defined as the earliest time when processor *p*_*j*_ will be available to begin executing task *T*_*i*_. Hence,

(4) EST(Ti,pj)={o,if  Ti=Tentry    max⁡⁡{Pav(Ti,pj),α},

where, *α* = max⁡_*T*_*j*_∈ *pred* (*T*_*i*_)_(*EFT*(*T*_*j*_, *p*_*k*_) + tr⁡_*ji*_

(5) $$\begin{array}{c} EFT\left(T_{i},p_{j}\right)=w_{ij}+EST\left(T_{i},p_{j}\right). \end{array}$$

Note that the Actual Start Time and Actual Finish Time of a task *T*_*i*_ on a processor *p*_*j*_, denoted as *AST *(*T*_*i*_, *p*_*j*_) and *AFT *(*T*_*i*_, *p*_*j*_) can be different from its earliest start *EST *(*T*_*i*_, *p*_*j*_) and finish *EFT *(*T*_*i*_, *p*_*j*_) times, if the actual finish time of another task *T*_*k*_ scheduled on the same processor is later than its *EST *(*T*_*k*_, *p*_*j*_) [44].

**Figure 1:**
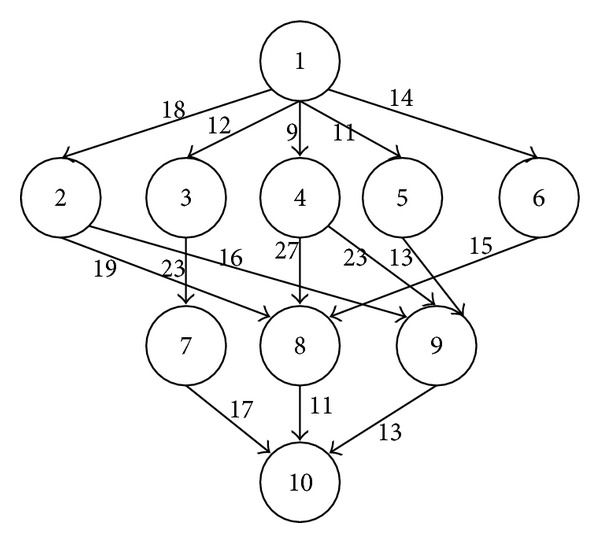
An example of workflow (given in [17]) with the task numbers *T*_*i*_ inside nodes and values of *d*_*ij*_ function next to the corresponding edges.

Figure 1 depicts a workflow application with 10 tasks, and the Table 2 provides its details (given in [17]). The values presented in the last column of the table represent the priority of the tasks. The priority of task *T*_*i*_ represented by Pr(*T*_*i*_) is computed recursively by traversing the DAG upward starting from the exit task *T*_exit_ as follows (6):

(6) Pr(Ti)={w¯Texit,if  Ti=Texitw¯i  +β,otherwise,

where, $\beta =\max_{T_{j}\;\in \;succ\;(T_{i})}\left\{{\bar{Tr}}_{ij}\;+\;\text{Pr}\left(T_{j}\right)\right\}$.

### 3.3. QoS Parameter Models

#### 3.3.1. Energy Model

Among the main system-level energy-saving techniques, Dynamic Voltage Scaling (DVS) operates on a simple principle: decreases the supply voltage (and so the clock frequency) to the CPU so as to consume less power.

In this work, we use a model of energy derived from the power consumption model in digital complementary metal-oxide semiconductor (CMOS) logic circuits [44]. Under the dynamic power model, the processor power is dominated by the dynamic power which is given by:

(7) $$\begin{array}{c} {\;P}_{\text{dynamic}}=AC_{ef}v^{2}f, \end{array}$$

where *A* is the number of switches per clock cycle, *C*_ef_ denotes the effective charged capacitance, *v* is the supply voltage, and *f* denotes the operational frequency. Equation (7) shows that the supply voltage is the dominant factor; hence, its reduction would be most influential to lower power consumption.

The energy consumption of the execution of a workflow application used in this paper is defined as:

(8) Ec=∑i=1nACefvi2fiwri∗=∑i=1nγvi2fiwri∗,

where *γ* = *AC*_ef_ is assumed constant for a given machine; *v*_*i*_, *f*_*i*_ are the voltage supply and frequency of the processor on which task *T*_*i*_ is executed, respectively, and *wr*_i_*is the real completion time of task *T*_*i*_ on the scheduled processor. In the idle time, the processor turns into sleep mode and thus the voltage supply and relative frequency are at the lowest level. So, the energy consumption during idle periods of processors is defined as:

(9) $$\begin{array}{c} E_{i}=\sum_{j=1}^{m}\;\;\sum_{{\text{idle}}_{jk}\in \;{\text{IDLE}}_{j}}\gamma v_{\min j}^{2}\;f_{\min j}\;L_{jk}, \end{array}$$

where IDLE_*j*_ is the set of idling slots on machine *p*_*j*_, *v*_min⁡*j*_(*f*_min⁡*j*_) is the lowest supply voltage (frequency) on *p*_*j*_, and *L*_*jk*_ is the amount of idling time for idle_*jk*_. Then the total energy *E*_total_ utilized by the cloud system for completion of the workflow application can be defined as follows:

(10) $$\begin{array}{c} E_{\text{total}}=E_{c}{+E}_{i}. \end{array}$$

#### 3.3.2. Time Model

The completion time *T*_total_ is the makespan from the user submitting a workflow until receiving the results. It is defined as the actual finish time of the exit task. It is calculated in equation 11 and consists of the workflow execution time and network transmission time. The execution time depends on both the workload and system performance. The network transmission time depends on both the network latency and the input data size

(11) $$\begin{array}{c} T_{\text{total}}=\max\text{AFT}\left(T_{\text{exit}}\right). \end{array}$$

#### 3.3.3. Cost Model

As result of the marketization characteristic of current services, most cloud providers have set a price for their services. They have fixed the price for transferring basic data unit (e.g., per MB) between two services and the price for processing basic time units (e.g., per hour). The cost *C*_total_ of running a cloud workflow is defined in Formula (12). It consists of processing cost *C*_ex_ and data transfers cost *C*_tr⁡_:

(12) $$\begin{array}{c} C_{\text{total}}=C_{\text{ex}\;}+C_{\mathrm{tr}}. \end{array}$$

The processing cost for a given task *T*_*i*_ depends on the real completion time of *T*_*i*_ on the scheduled processor (*wr*_i_*), and the hourly price of this processor (ec_*i*_). Thus, *C*_ex_ is given by:

(13) $$\begin{array}{c} C_{\text{ex}}=\sum_{i=1}^{n}{wr}_{i}^{*}*{\text{ec}}_{i}. \end{array}$$

The data transfer cost (*C*_tr⁡_) is described as follows:

(14) $$\begin{array}{c} C_{\mathrm{tr}}=\sum_{i=1}^{n}\;\sum_{j=1}^{n}d_{ij}*{\text{trc}}_{ij}, \end{array}$$

where *d*_*ij*_ characterizes the output file size from task *T*_*i*_ to task *T*_*j*_; and tr⁡*c*_*ij*_ represents the cost of communication from the processor where *T*_*i*_ is mapped to another processor where *T*_*j*_ is mapped. The cost of communication is added to the overall cost only when two tasks have data dependency between them, (i.e, *d*_*ij*_> 0). For two or more tasks running on the same processor, the transfer cost is neglected.

#### 3.3.4. Scheduling Model

Given (1) A cloud provider that offers a set *P* of *m* heterogeneous processors and (2) a user workflow application composed of a set *T* of *n* tasks that have to be executed on these processors. The workflow scheduling problem is to construct a mapping *M* of tasks to processors (without violating precedence constraints) that minimizes the following conflicting objectives: makespan, cost, and energy consumption as low as possible. Therefore the workflow scheduling problem can be formulated as a mathematical optimization problem:

(15) $$\begin{array}{c} \text{Makespan:}\;\text{Minimize}\;\;T_{\text{total}}\left(M\right)\\ \text{Cost:}\;\text{Minimize}\;\;C_{\text{total}}\left(M\right)\\ \text{Energy:}\;\text{Minimize}\;E_{\text{total}}\left(M\right). \end{array}$$

## 4. Workflow Scheduling Based on Discrete Particle Swarm Optimization

This section starts with a brief overview on multi-objective combinatorial optimization and Particle swarm optimization algorithm. Afterwards, our new Multi-Objective Discrete Swarm Optimization combined with DVFS technique will be presented.

### 4.1. Multi-Objective Optimization

A Multi-objective Optimization Problem (MOP) with *m* decision variables and *n* objectives can be formally defined as:

(16) $$\begin{array}{c} \mathrm{Min}\left(y=f\left(x\right)=\left[f_{1}\left(x\right),\ldots ,f_{n}\left(x\right)\right]\right), \end{array}$$

where *x* = (*x*_1_,…, *x*_*m*_) ∈ *X* is an *m*-dimensional decision vector,  *X* is the search space, *y* = (*y*_1_,…, *y*_*n*_) ∈ *Y* is the objective vector and Y the objective-space.

In general MOP, there is no single optimal solution with regards to all objectives. This is also the case for the multi-objective optimization problem addressed in this paper. As given in (15), there are three conflicting objectives: minimizing execution time, minimizing execution cost and minimizing energy consumption. In such problems, the desired solution is considered to be the set of potential solutions which are all optimal in some objectives. This set is known as the Pareto optimal set. We provide some definitions of the Pareto concepts used in MOP as follows: (without loss of generality we suppose that the objectives are to be minimized):

(i)   *Pareto dominance*. For two decision vectors *x* ^1^ and *x* ^2^, dominance (denoted by ≺) is defined as follows:
  (17) $$\begin{array}{c} x^{1}≺x^{2\;}\Leftrightarrow \forall i\;f_{i}\left(x^{1}\right)\leq f_{i}\left(x^{2}\right)\wedge \exists jf_{j}\left(x^{1}\right)<f_{j}\left(x^{2}\right). \end{array}$$
  The decision vector *x* ^1^is said to dominate *x* ^2^ if and only if, *x* ^1^ is as good as *x* ^2^ considering all objectives and *x* ^1^ is strictly better than *x* ^2^ in at least one objective.
(ii) *Pareto optimally*. A decision vector *x* ^1^ is said to be Pareto optimal if and only if
  (18) $$\begin{array}{c} ∄x^{2}\in X:x^{2}≺x^{1}. \end{array}$$
(iii) *Pareto optimal set*. The Pareto optimal set *P*_*S*_ is the set of all Pareto optimal decision vectors.
  (19) $$\begin{array}{c} P_{S}\;=\left\{x^{1}\in X,\;|\;∄x^{2}\in X:x^{2}≺x^{1}\right\}. \end{array}$$
(iv) *Pareto optimal front*. The Pareto optimal front *P*_*F*_ is the image of the Pareto optimal set in the objective space.
  (20) $$\begin{array}{c} P_{F}=\left\{f\left(x\right)=\left(f_{1}\left(x\right),\ldots ,f_{n}\left(x\right)\right)\;|\;x\in P_{s}\right\}. \end{array}$$
 
*x* ^1^ is said to be non-dominated regarding a given set if *x* ^1^ is not dominated by any decision vectors in the set.

The pareto optimal decision vector cannot be enhanced in any objective without causing degradation in at least another objective. A decision vector is said to be Pareto optimal when it is not dominated in the whole search space.

### 4.2. Particle Swarm Optimisation

#### 4.2.1. The Standard PSO

PSO is a population-based stochastic optimization technique developed by Kennedy and Eberhart in 1995 [47]. It is inspired by the social behavior of insect colonies, bird flocks, fish schools and other animal societies. It is also related to evolutionary computation. PSO has attracted significant attention from many researchers due to both its simplicity of use and optimization via social behavior. In fact, PSO has good performance, requires low computational cost. It is effective and easy to implement as it uses numerical encoding. 

A particle in PSO is analogous to a fish or bird moving in the *D*-dimensional search space. All particles have fitness values indicating their performances, which are problem specific, and velocities which direct the flight of particles. Each particle position at any given time is influenced by both its best position called *pBest* and the position of the best particle in a problem space referred to as *gBest*. Therefore particles tend to fly towards a better search area during the search process. A particle status on the search space is characterized by two elements, namely its velocity and position, which are updated in every generation as follows:

(21) Vik+1=ωVik+c1r1(pBesti−Xik)+c2r2(gBest−Xik),Xik+1=Xik+Vik+1,

where *V*_*i*_^*k*+1^ is the velocity of particle *i* at iteration *k* + 1, *V*_*i*_^*k*^ is the velocity of particle *i* at iteration *k*, *ω* is the inertia weight, *c*_1_ and *c*_2_ are the acceleration coefficients (cognitive and social coefficients), *r*_1_ and *r*_2_ are the random numbers between 0 and 1, *X*_*i*_^*k*^ is the current position of particle *i* at the *k*th iteration, *pBest*_*i*_ is the best previous position of the *i*th particle, *gBest* is the position of best particle in the swarm, and *X*_*i*_^*k*+1^ is the position of *i*th particle at *k* + 1 iteration.

The procedure for standard PSO is as follows:

1. Initialize a population of particles with random positions and velocities in the search space.
2. Evaluate the objective values of all particles, set *pBest* of each particle equal to its current position, and set *gBest* equal to the position of the best initial particle.
3. Update the velocity and the position of each particle according to (21).
4. Map the position of each particle in the solution space and evaluate its fitness value according to the desired optimization fitness function.
5. For each particle, compare its current objective value with its *pBest* value. If the current value is better, then update *p*Best with the current position and objective value.
6. Determine the best particle of the current whole population with the best objective value. If the objective value is better than that of *gBest*, then update *g*Best with the current best particle.
7. If the stopping criterion is met, then output *gBest* and its objective value; otherwise, go to Step (2).

The original design of PSO is appropriate for finding solutions to continuous optimization problems. However, as the workflow scheduling discussed in this paper is both a discrete and multi objective problem in nature, we propose an effective approach to address this problem by using a discrete version of the Multi-Objective PSO (MODPSO) combined with DVFS technique. The key issues of our approach consist of: (1) defining the position and velocity of the particles based on the features of discrete variables of the workflow scheduling; (2) solving the multi-objective aspect of the problem by modifying PSO so as to generate a set of non-dominated solutions satisfying the different objectives under consideration instead of one solution.

#### 4.2.2. Handling Workflow Scheduling Using DVFS-MODPSO

In general, a workflow scheduling can be defined by a set of triplets *M* = [〈*T*_*i*_, *P*_*j*_, *L*_*k*_〉](*i* ∈ [1, *n*], *j* ∈ [1, *m*], *k* ∈ [1, *L*(*j*)]),  *n* is the number of workflow tasks to be scheduled, *m* is the number of processors available in the cloud environment and *L*(*j*) is the number of operating points (VSLs) of the *j*th processor.

For the sake of clarity, the variables and rules of DVFS-MODPSO for solving workflow scheduling can be depicted as follows:

 The position of a particle represents a feasible solution to the scheduling problem. It consists of a set of 〈task(*T*_*i*_),  service  (*P*_*j*_),  VSl  (*L*_*k*_)〉 triplets. Each triplet means that a task (*T*_*i*_) is assigned to a processor (*P*_*j*_) with a voltage scaling level (*L*_*k*_). It also indicates that the position satisfies the precedence constraint between tasks.
 The process of generating a new position for a selected particle in the swarm is depicted in the following formulas:
  (22) Vik+1=Vik⊕((R1⊗(pBesti⊖Xik))    ⊕(R2⊗(gBest⊖Xik)))
  (23) $$\begin{array}{c} X_{i}^{k+1}=X_{i}^{k}⊕V_{i}^{k+1}. \end{array}$$
 The operator definitions are as follows:
(i) *The substract operator *(⊖). the difference between two particle positions, designated as *x*1 and *x*2, is defined as a set of triplets in which each triplet 〈task, service, VSL〉 shows whether the contents of the corresponding elements in *x*1 are different from those of *x*2 or not. If so, that triplet gets its values (service and VSL) from the position that has the lowest value of the VSL. For those triplets that have the same content in *x*1 and *x*2, their corresponding VSLs are decreased. (Note that the scaling of VSL makes fluctuations on the energy, makespan and cost).
(ii) *The multiply operator *(⊗). the multiplication between number and velocity is defined as a set of triplets, where: a threshold *α* ∈ [0, 1] is defined, a random number *r* is generated for each triplet 〈task, service, VSL〉; compare *r* and *α*: when *r* ≥ *α*, decrease the triplet VSL, otherwise, increase it. This operator adds the exploration ability to the algorithm.
(iii) *The add operator *(⊕). the addition of two positions is defined as the reservation of the dominated one.

The Pseudocode 1 outlines the general steps of the DVFS-MODPSO algorithm. 

**Pseudocode 1:**
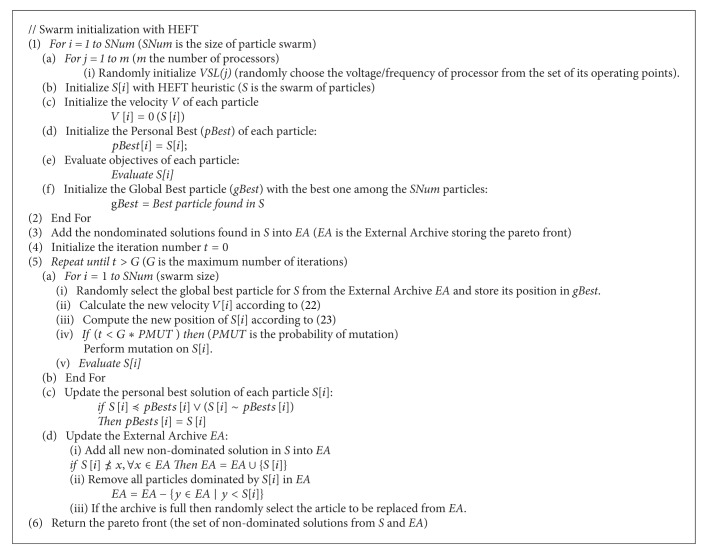
DVFS-MODPSO based workflow scheduling.

The algorithm begins by initializing the positions and velocities of particles. To obtain the position of a particle, the VSL (voltage and frequency) of each resource is randomly initialized firstly then the HEFT algorithm is applied to generate a feasible and efficient solution minimizing the makespan. The process is repeated several times to initialize the positions of all particles of the swarm. Initially, the velocity*V* and the best position for each particle *pBest* are attributed as the particle itself. The algorithm maintains an external archive EA to store non-dominated particles found after the evaluation process (based on the pareto dominance using the objectives mentioned in 3.3). After all these initializations, in the main loop of the algorithm, the new velocity and position of each particle are calculated respectively after selecting the best overall position in the external archive and eventually perform a mutation, then the particle is evaluated and its corresponding *pBest* is updated. The external archive is updated after every iteration. Once the termination condition is reached, the external archive containing the Pareto front is returned as the result.

## 5. Experimental Evaluations

In this section, we describe the overall setup of our experimentation effort and the results we have obtained from it to validate the new proposed approach.

**Figure 2:**
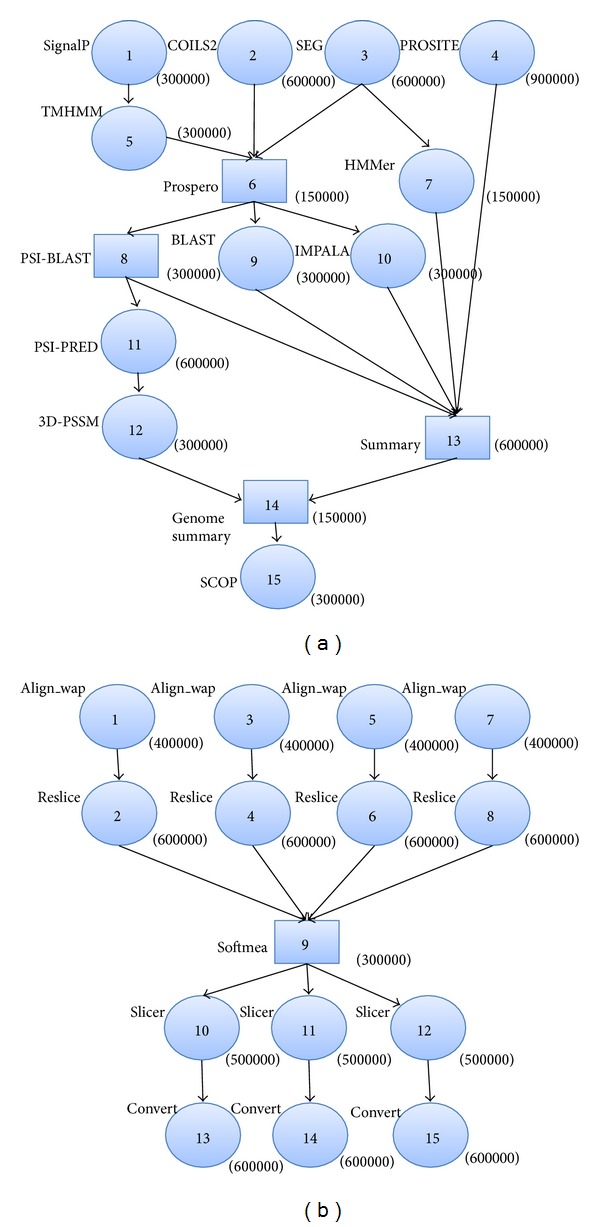
Parallel and Hybrid workflows.

In our experiments, we simulate a synthetic workflow application described in [17] (see Figure 1) and two common workflow structures: parallel and hybrid structures. We chose two well known real world applications, namely a neuro-science workflow [48] for our parallel application and a protein annotation workflow [49] developed by London e-Science Centre for our hybrid workflow application.  Figure 2 shows their simplified representations. The number indicated in the parentheses next to each node represents the length of the task (the number of instructions to execute) in Millions of Instructions (MI). The input and output files of each task range from 10 MB to 1024 MB.

We have performed experiments on 3, 4, 5, 6 and 12 resources whose characteristics are shown in Table 1. We choose the pricing model associated with Amazon EC2 (http://aws.amazon.com/ec2) for the processing costs of the different classes of resources, and the pricing model given by Amazon CloudFront (http://aws.amazon.com/cloudfront/) for the costs of transferring data unit between resources. 

As for *DVFS-MODPSO*, the parameter settings are: Swarm size, *SNum* = 50 particles and the maximum number of iterations, *G* = 100.

We evaluated the performance of our proposed *DVFS-MODPSO* on all the workflow instances described previously. Due to the lack of works considering both the heterogeneous configuration of our cloud and the three QoS metrics (makespan, cost, energy) at the same time, we compared our results with those of the HEFT heuristic we have implemented. HEFT is one of the most widely used heuristics for DAGs in distributed heterogeneous computing systems which optimize the makespan. 

**Figure 3:**
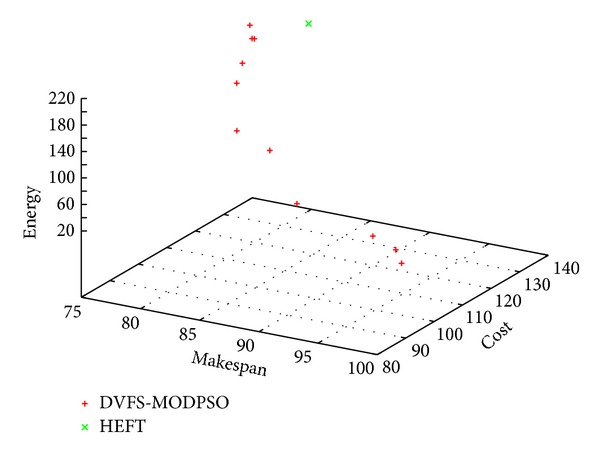
Obtained non-dominated solutions for the synthetic workflow.

**Figure 4:**
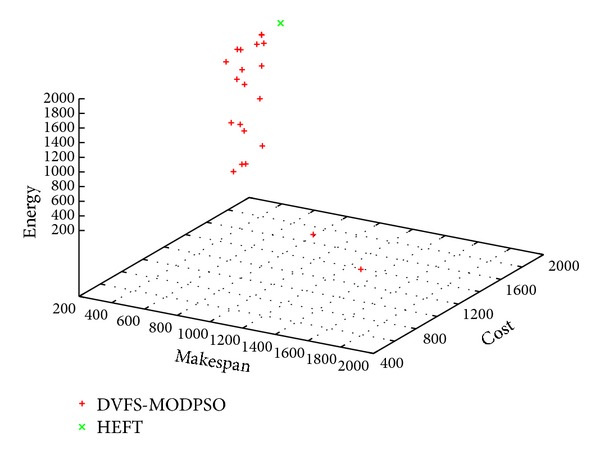
Obtained non-dominated solutions for the hybrid workflow.

**Figure 5:**
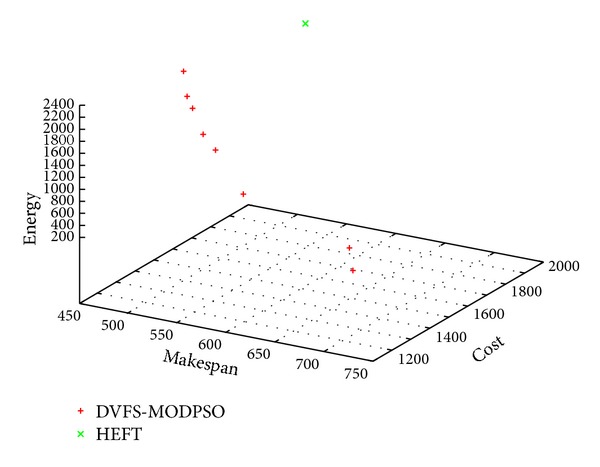
Obtained non-dominated solutions for the parallel workflow.

Figures 3, 4 and 5 show sample Pareto fronts obtained with the *DVFS-MODPSO* and the solution computed by HEFT for the synthetic workflow, the hybrid workflow and the parallel workflow, respectively.

As can be seen from these figures, unlike the HEFT algorithm which gives one solution, our proposed approach provides a set of non dominated solutions. These results illustrate the basic multi-objective definitions provided in Section 4.1.

Now, for comparing these two approaches, and in order to analyze the effectiveness of our proposition, in terms of the values of makespan, cost and energy consumption, we have been inspired by the methodology described in [43] where we compare the solution provided by *HEFT* to only one solution of the Pareto front set computed by our proposed multi-objective *DVFS-MODPSO.*

The evaluation process follows the next steps. For each workflow instance, we perform a first resolution using *HEFT* in order to get one solution *S*. After, a second resolution is computed using our proposed approach to obtain a set *EA* of Pareto solutions. Next, we select one solution *S*′ from the set *EA* of Pareto solutions. This solution is the closest one to *S* in the sense of Euclidean distance. Finally, a comparison is done between *S* and *S*′.

**Figure 6:**
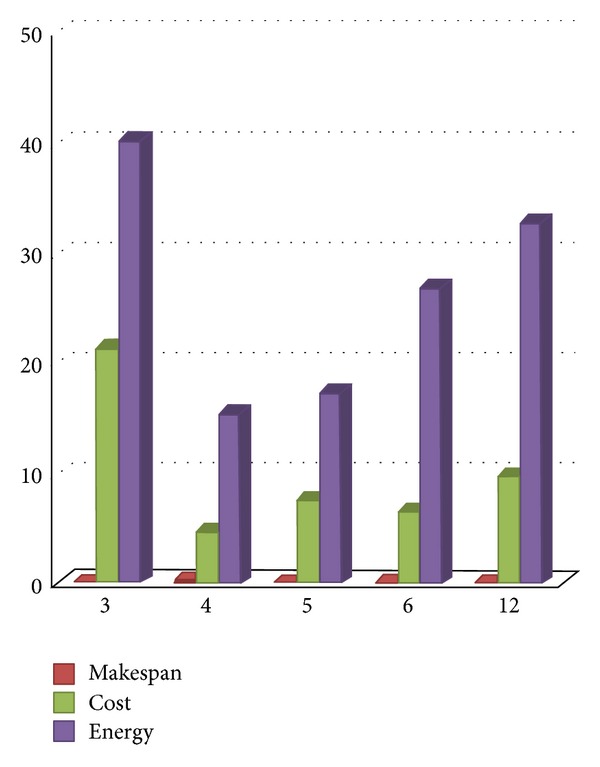
Gain over *HEFT* (%) according to the number of processors.

**Figure 7:**
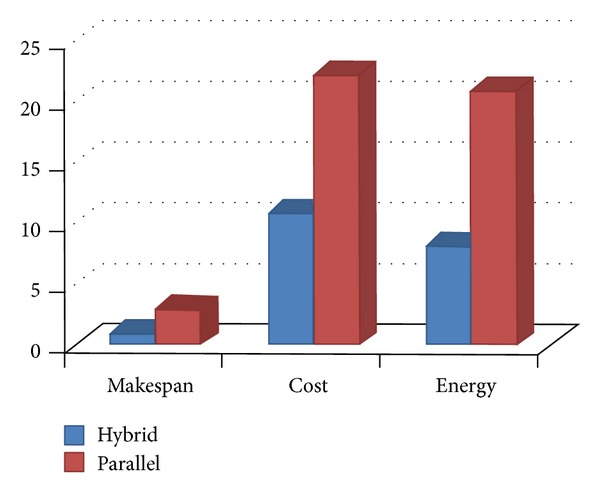
Improvement according to real world applications.

**Figure 8:**
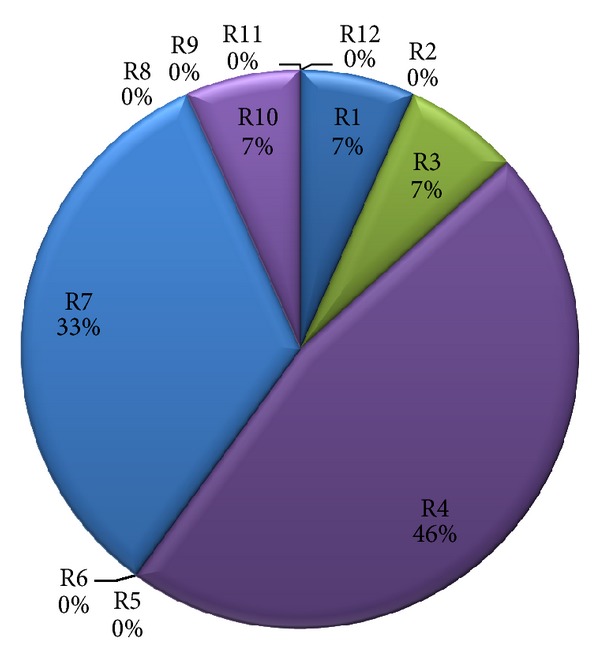
Resource loads for synthetic workflow.

**Figure 9:**
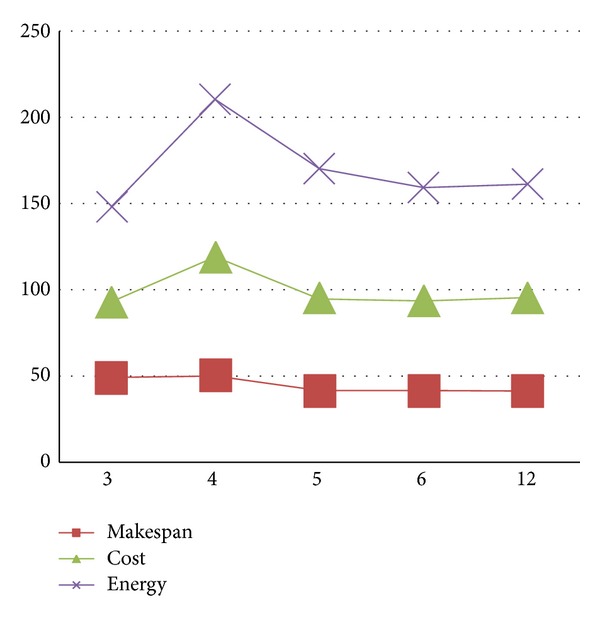
QoS metrics evolutions according to resource numbers for the synthetic workflow.

The different results are displayed on Figures 6, 7, 8, and 9 and on Tables 3 and 4. They show the improvement achieved by our proposed approach according to the structure of the workflow applications and the number of processors.

Table 3 and Figure 6 show that our proposed approach improves on average the result obtained by HEFT for the synthetic workflow instance. The makespan is reduced by 0.05%, the cost is reduced by 9.93% and the energy consumption is reduced by 26.40%. Figure 6 shows detailed improvements of the proposed approach according to the number of processors. 

Table 4 and Figure 7 illustrate the gain obtained by our approach according to the kind of real world applications. Table 4 also shows the detailed improvements when scaling the number of processors. As can be seen, the QoS metrics are improved over *HEFT* in average by 0.95% for the makespan, 10.8% for cost and 8.12% for the energy consumption when using the hybrid workflow application, and average improvements of 2.95% for makespan, 22.15% for cost and 20.9% for energy consumption when using the parallel workflow.

These evaluations confirm the results obtained from the synthetic workflow. This means that by using our *DVFS-MODPSO*, we are able to improve not only the cost and energy but also the makespan on which HEFT algorithm is supposed to provide good results. These results remain true for all kind of workflow applications.

In our experiments, we limited the number of resources to 12, as we found that, for these kinds of workflow applications, some resources are not used at all.  Figure 8 shows the resource loads when scheduling the synthetic workflow on 12 resources. Furthermore, even though the number of resources increased, the total cost and total energy consumption could not always decrease as illustrated in Figure 9. From this figure, we can see that the QoS metrics (1) firstly decreases as the number of resources increases until achieving the number 6. This can be explained by the fact that when increasing the number of resources, there are fewer tasks executed in a resource, therefore, tasks can extend their execution times and the resource have more of chances to scale down their voltages and frequencies which can be very effective in reducing total energy consumption. (2) After achieving 6 resources, the QoS metrics begin to rise; the reason for this is that time executions are dominated by interprocessors communications, hence decreasing the opportunities for scaling down voltages and frequencies of resources. Consequently, the threshold numbers of resources that minimized the QoS metrics could be obtained. 

## 6. Conclusion

In this paper, we propose a new algorithm called DVFS-Multi-Objective Discrete Particle Swarm Optimization (*DVFS-MODPSO*) for workflow scheduling in distributed environments such as cloud computing infrastructures. *DVFS-MODPSO* simultaneously optimizes several conflicting objectives namely, the makespan, cost and energy in a discrete space. It produces a set of non-dominated solutions, thus providing more flexibility for users to assess their preferences and select a schedule that meets their QoS requirements. Our approach exploits the heterogeneity and the marketization of cloud resources in order to find solutions that optimize makespan and cost. Furthermore, it uses the Dynamic Voltage and Frequency Scaling (*DVFS*) technique to reduce energy consumption.

We have evaluated our algorithm by simulating it with both synthetic and real world scientific workflow applications having different structures. The results show that the proposed *DVFS-MODPSO* is able to produce a set of good Pareto optimal solutions. The results also show that our approach provides significant improvement not only in terms of the cost and the energy consumption but also in term of the makespan for which HEFT algorithm is supposed to give better results.

Multi-objective optimization in cloud workflow scheduling is not a mature domain. Most of the existing works attempt to minimize either the makespan or cost. However, we plan to consider other objectives such as reliability, security in addition to the energy consumption. We also intend to apply our algorithm in a real world cloud or integrate it in existing cloud toolkits such as Cloudbus for comparing with other algorithms.

## Figures and Tables

**Table 1:** Voltage Scaling Levels and relative speeds of processors.

VSL.	P1: TURION MT-34			P2: OMAP			P3: PENTIUM M		
	Volt.	Freq.	R. Speed	Volt.	Freq.	R. Speed	Volt.	Freq.	R. Speed
	(V)	(Ghz)	(%)	(V)	(Ghz)	(%)	(V)	(Ghz)	(%)
1	1.2	1.8	100	1.35	0.6	100	1.48	1.4	100
2	1.15	1.6	88.88	1.27	0.55	91.66	1.44	1.2	96.76
3	1.1	1.4	77.77	1.2	0.5	83.33	1.31	1	88.14
4	1.05	1.2	66.66	1	0.25	41.66	1.18	0.8	79.51
5	1	1	55.555	0.9	0.13	20.83	0.96	0.6	64.42
6	0.9	0.8	44.44						

Cost	ec = 1.14			ec = 0.57			ec = 0.76		

**Table 2:** Task execution times and priorities.

Task (*T*_*i*_)	*p*_1_	*p*_2_	*p*_3_	${\bar{\omega }}_{i}$	Priority (Pr(*T*_*i*_))
1	14	16	9	13	108.000
2	13	19	18	16.67	77.000
3	11	13	19	14.33	80.000
4	13	8	17	12.67	80.000
5	12	13	10	11.67	69.000
6	13	16	9	12.67	63.333
7	07	15	11	11	42.667
8	5	11	14	10	35.667
9	18	12	20	16.67	44.333
10	21	7	16	14.67	14.667

**Table 3:** Improvement for synthetic workflow.

Num. of proc.	Gain over Heft (%)		
	Makespan	Cost	Energy
3	0	21.26	40.08
4	0.22	4.64	15.35
5	0	7.56	17.26
6	0.03	6.52	26.75
12	0	9.69	32.55

Average	0.05	9.93	26.40

**Table 4:** Improvement according to real world applications.

Appli.	Num. of proc	Gain over Heft (%)		
		Makespan	Cost	Energy
Hybrid	3	4.05	11.55	04.02
	4	0.28	05.66	05.07
	5	0.00	13.47	11.24
	6	0.00	01.17	12.65
	12	0.43	22.14	07.61
	Average	**0.95**	**10.80**	**08.12**

Parallel	3	2.35	56.40	25.52
	4	11.47	20.94	32.71
	5	0.00	21.42	11.66
	6	0.93	11.70	31.54
	12	0.00	0.31	3.06
	Average	**2.95**	**22.15**	**20.9**
